# Smart nanomaterial and nanocomposite with advanced agrochemical activities

**DOI:** 10.1186/s11671-021-03612-0

**Published:** 2021-10-18

**Authors:** Antul Kumar, Anuj Choudhary, Harmanjot Kaur, Sahil Mehta, Azamal Husen

**Affiliations:** 1 Department of Botany, Punjab Agricultural University, Ludhiana, 141004 India; 2 International Centre for Genetic Engineering and Biotechnology, Aruna Asaf Ali Marg, New Delhi, 110067 India; 3 Wolaita Sodo University, Wolaita, Ethiopia

**Keywords:** Conventional agriculture, Agrochemicals, Nanomaterial, Crop improvement, Sustainable

## Abstract

Conventional agriculture solely depends upon highly chemical compounds that have negatively ill-affected the health of every living being and the entire ecosystem. Thus, the smart delivery of desired components in a sustainable manner to crop plants is the primary need to maintain soil health in the upcoming years. The premature loss of growth-promoting ingredients and their extended degradation in the soil increases the demand for reliable novel techniques. In this regard, nanotechnology has offered to revolutionize the agrotechnological area that has the imminent potential over conventional agriculture and helps to reform resilient cropping systems withholding prominent food security for the ever-growing world population. Further, in-depth investigation on plant-nanoparticles interactions creates new avenues toward crop improvement via enhanced crop yield, disease resistance, and efficient nutrient utilization. The incorporation of nanomaterial with smart agrochemical activities and establishing a new framework relevant to enhance efficacy ultimately help to address the social acceptance, potential hazards, and management issues in the future. Here, we highlight the role of nanomaterial or nanocomposite as a sustainable as well stable alternative in crop protection and production. Additionally, the information on the controlled released system, role in interaction with soil and microbiome, the promising role of nanocomposite as nanopesticide, nanoherbicide, nanofertilizer, and their limitations in agrochemical activities are discussed in the present review.

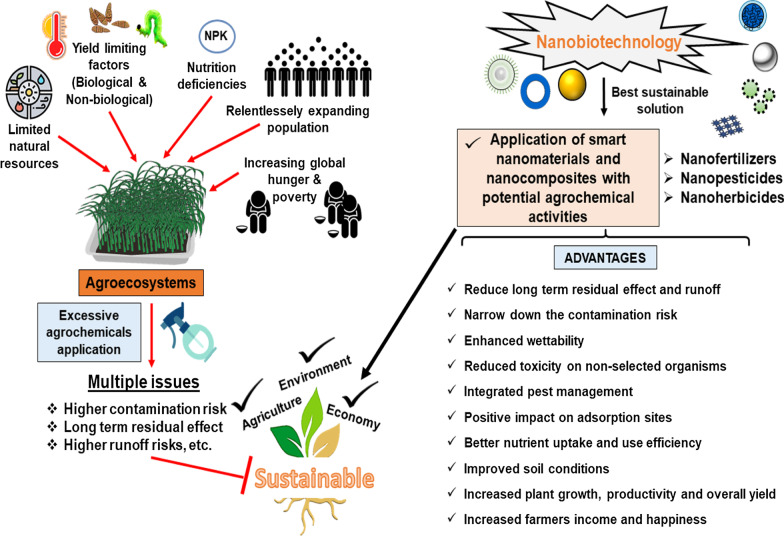

## Introduction

Globally, people are employed in agriculture for the cultivation of fundamental food crops and various essential forms of products such as fibers, fuels, fodders, and raw materials. Limited resources and an exponentially growing population, which is estimated to mark 9.6 billion by 2050, enforce the areas derived demanding the elaboration of very sustainable agriculture while permitting declination of global hunger and poverty [1, 2]. To fulfill this demand of relentlessly expanding population, there is an urgent prerequisite to enhance food production by more than 50% [2, 3]. Due to the limited number of natural resources (water, land, soil, forest, etc.) and ceiling in crop productivity, there is a huge demand for effective agricultural approaches that are viable and liable economically and eco-friendly. To overcome these dilemmas, synthetic agrochemicals (herbicides, insecticides, fungicides, and fertilizers) have been developed and used to increase agricultural yields [4, 5]. However, the application of such agrochemicals had been instrumental for elevating food quality and quantity in past decades to evaluate the long-term ill effect of such agrochemicals on soil health and the ecosystem [6]. However, research on nanoparticle application as chemical alternatives for utility in the agriculture sector has become enhancing popularity over the past decade, later referred to as nanoagrochemicals [7]. The intentional and directional delivery in the environment, nanoagrochemicals may be considered specific in terms of expectable environmental issues, as they would represent the single diffuse cause of engineered nanoparticles (NPs) [8, 9]. Given this, one such initiative taken is the forefront of smart nanomaterials for revolutionizing current agriculture practices that contain good reactivity due to their substantial surface area to volume ratio and exceptional physicochemical characteristics that offer the novel advantage of modification according to increasing demand [2].

**Fig. 1:**
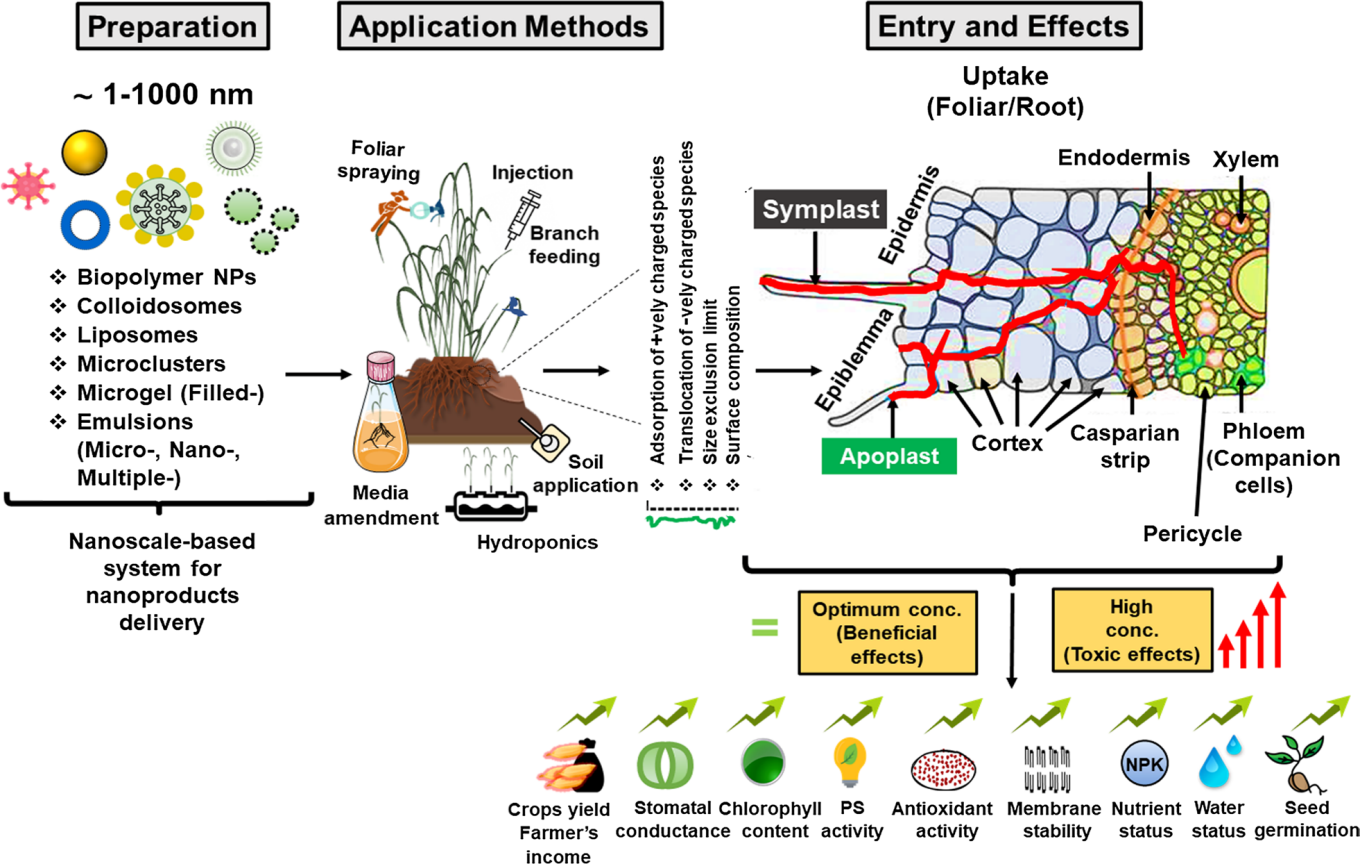
Diagrammatic illustration of nanoparticles transport, and their interactions in crop plant

Modern agriculture is renovating into sustainable agriculture with the use of these modern age materials that are empowering to attain maximum output from limited resources [10]. Generally, agrochemical is essential to increase crop productivity but contrary, their application decline soil fertility by hindering soil mineral balance [11]. Moreover, the direct foliar or sprayed application can be cost-effective and very high, which run off and need to be controlled [12]. The nanomaterials-based chemicals developed in agriculture regulate nutrient depletion rate, yield reduction, input cost for crop raising, protection, production, and minimizing post-harvest loss [3]. Nanocomposites have become a key component of nanomaterials for scrutinizing and stimulating the plant life cycle because of their intrinsic unique thermal, electrical, chemical, and mechanical properties. The translocation in size-dependent lies in the range of 0.1–1000 nm within plant parts and altered according to surface compositions, a charge of NPs (highly negatively charged shows more translocation), and plant size exclusion limit [10, 13]. These routes of penetration are confirmed via different in vitro (Filter paper, hydroponics, agar media, Hoagland solution, Mursashige and Skoog media, nutrient solution) and in vivo (foliar uptake, branch feeding, trunk injection, and root uptake) experiments using nanopesticide, nanoherbicide, nanoherbicides, and nanogrowth-promoting compounds [2, 9]. However, in certain cases the size exclusion is high so, it’s difficult to limits the specific passage and concentration that affect the growth phase of plants both positively and negatively (Fig. 1).

Many successful examples of utilizing smart nanomaterial in agriculture have been reported in recent years including multi-walled carbon nanotubes [5, 14], metal-based nanocomposites [15], silver inhibits fungus germination [16], and many more. This new-age nanoformulation has the potential to fine-tune the physiology just entering the soil–plant complex that can be solely exploited to spotify the lateral effect [17].

The nanoparticle-based products (NMs) including smart agrochemical delivery systems having nanocomposites as chief ingredients are being constantly developed. Much intensive research is still required to achieve the practical advantages of nanoagrochemicals with improved working design, regulation of commercialization, and risk assessment of nanofertilizer, nanopesticide, and nanoherbicide [18, 19]. New crop cultivars, that can sustain heat, drought, salinity, and other unresolved challenges in farming systems disturb the whole spectrum of major cultivation practices worldwide. Moreover, it is expected that the implementation of NMs in the natural environment decline the chemicals-based hazardous level [12]. We surely believe, their application in agriculture will narrow down the gap between sustainable and chemical-based agriculture systems. Besides this, it boosts food production and quality globally in an eco-friendly manner by resolving water and soil contamination [20]. Thus, practically they could provide novel avenues regarding developing new NMs-based products [14]. Conventional agrochemical has offered numerous drawbacks regarding the non-selectively and adsorption rate of active ingredients (AIs).

It has been reported that more than 99.9% pesticides are failed to be delivered at target sites and cause a hazardous impact on the health of the soil, water, air with enhances pathogenic resistance and biodiversity loss [12, 21, 22]. Overall, we aimed to highlight the current information on facts that nanomaterial or nanocomposite deliver an efficient solution to upgrade and advanced the agriculture innovations, food systems, sustainable crop protection, and production. Moreover, information on the controlled released system, role in interaction with soil and microbiome, the promising role of nanocomposite as nanopesticide, nanoherbicide, nanofertilizer, and limitation in agrochemical activities are also discussed in the present review.

## Nanostructure compounds with the controlled released system (CRS)

**Fig. 2:**
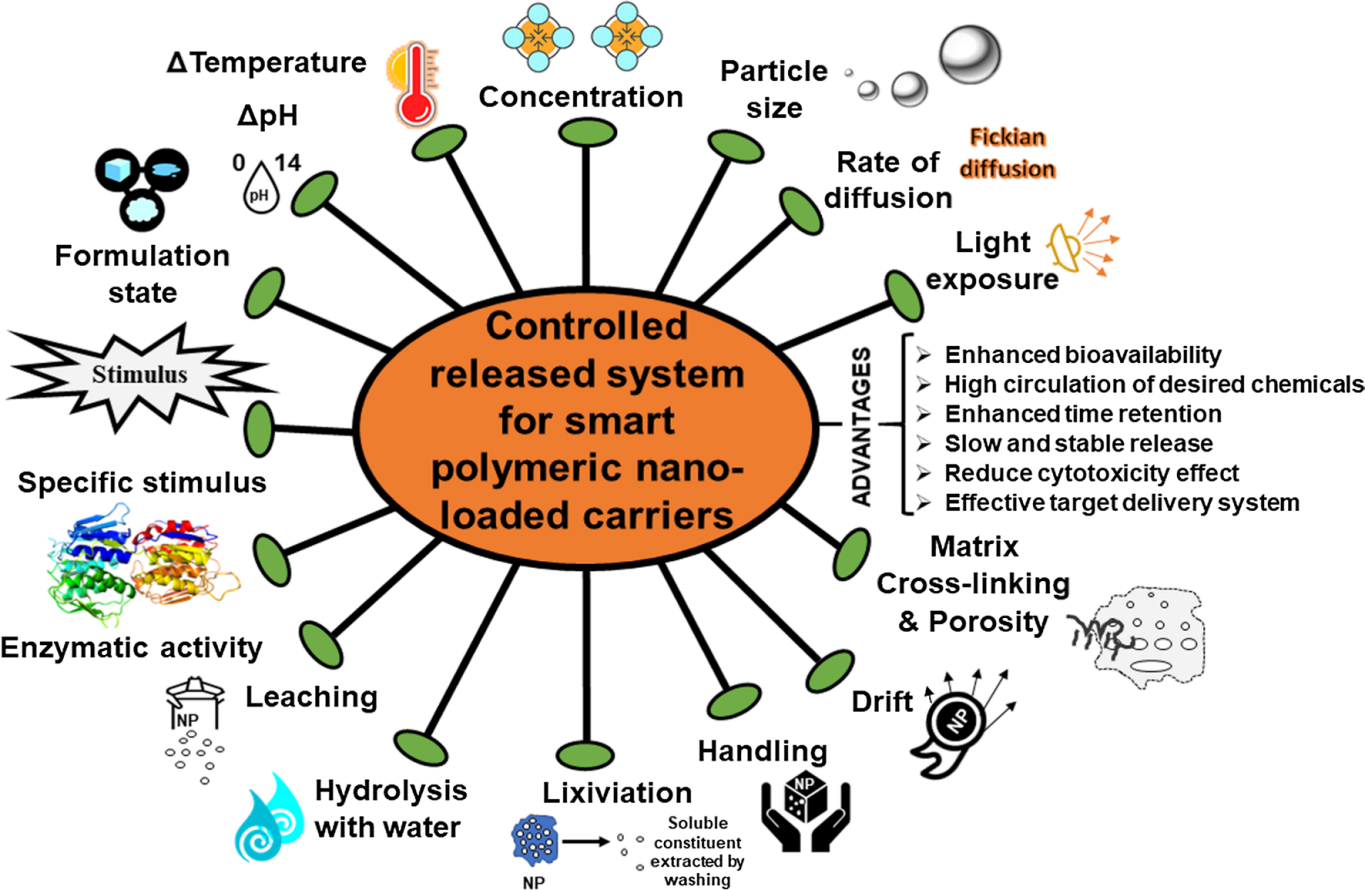
Types of nanoparticle delivery system

Due to several advantages over conventional chemical application approaches, many researchers have put forward the model of the controlled release system [15, 23–29] to offer substitutes to reduce environmental pollution. The controlled release (CR) allows efficient delivery of an AI more actively in soil and plant for the desired interval of time, resulting in the decreases of the amounts of agrochemicals used, energy, manpower, or other resources crucial to operate the application instruments as well as in enhancement in safety to humans who deal with their application [26, 29–32]. Additionally, CR shows many advantages over conventional methods including decrease phytotoxicity, reduce agrochemical loss due to volatilization, lixiviation, drift, improper handling, and degradation in soil and controlled delivery coincides with a suitable concentration in the plant to prevent unpredictable losses in form of evaporation, leaching and weather **(**Fig. 2) [16, 33].

Comprehensive characterization is a significant prerequisite to predict or explain the efficiency and behaviour of smart nano-loaded agrochemicals. In particular, retention of AIs, behaviour, composition and phase, zeta potential, and internal structure of polymeric nanocarriers, and their release in particle environment conditions are summarized as important properties [30, 34–36]. The rate of loading and release for AIs from nanocarriers plays a central role in predicting or assessing their efficacy. These can be evaluated by ingredients concentration remaining within polymeric matrix and amount of released ingredients [37, 38]. The mechanism of release can be achieved via different modes such as:

### Diffusion via relaxation/swelling of NPs

In the concentration gradient phenomena (or fickian diffusion), the release would occur at a high rate when nanocarriers are diluted using either concentrated or solid formulations even under irrigation or rainfall events. The diffusion can be slowdown by enhancing the nanoparticle size or enhancing the distance within media in which diffusion of AI occurs observed in poly lactic acid (PLA) loaded metazachlor [32, 39, 40]. Similarly, enhanced cross-linking has been suggested as an efficient method to delay diffusion by increasing the tortuosity or decreasing the porosity via the polymer matrix, as indicates by methomyl-loaded chitosan (azidobenzaldehyde-carboxymethyl) pesticide before and after polymer crosslinking [40–43].

### Burst release

The most commonly rapid release method in which AI release undesirably, if an initial high amount of AI is not favorable for the application of target. The phenomena would show enhance the concentration of AIs present near or on the surface of the NPs indicates high significant burst release. For example, PLA-loaded metazachlor (herbicides) nanocapsule or surface coating has been recommended to inhibits the initial rapid burst that is frequently noted for nanospheres [35].

### Degradation

Nanoparticle release can be triggered or accelerated by physical, chemical, and biological degradation that can be achieved by hydrolysis with water, light exposure, temperature, pH, specific stimulus, and enzymatic activities. For example, PLGA (Poly lactic co-glycolic acid) NPs show increased hydrolytic degradation with enhancing surface area- volume ratio for water, and their diffusion rate might be fine-tuned with appropriate nanocarriers [44]. Moreover, the mPEG (methoxy polyethylene glycol) incorporated in PLGA-NPs increases the degradation rate of NPs via enhanced hydrophilicity and ultimately accessibility for hydrolysis in hydrolytic degradation type. In enzymatic degradation, the events lead by the activities of phosphatases, glycosidases, and protease viz: PCL (poly(ε-caprolactone) degradation enhance with the activity of lipase activity [44]. Similarly, γ-PGA (poly (γ-glutamic acid) degradation mediated by γ-GTP (γ-glutamyl transpeptidase) is considered as a most common enzyme that causes rapid degradation [38]. In another study, zein nanoparticle shows rapid and extensive degradation and release of encapsulated ciprofloxacin antibiotic, in presence of trypsin enzyme than collagenase [37].

In some cases, stimuli-response release can be observed using photosensitive polymers such as micellar or UV (Ultraviolet) labile core–shell NPs were produced to PEG and nitrobenzyl to carboxymethyl chitosan. Thus, stimuli-based nanocomposite can intelligently react to the stimulus produced by the target or the adjoining environment that eventually triggers the AIs release to regulate the pest effectively [45, 46]. However, physical stability in some NPs altered by pH, when the polymer is weak basic or acidic such that electrostatic and charge will reliable on pH [40, 41, 47]. For instance, carboxymethyl cellulose and feather keratin were loaded with avermectin. The diffusion rate was observed to be faster at low pH (Fickian transport) and higher pH (non-Fickian) [46].

## Nanoformulations as a promising tool in an agricultural system

**Fig. 3:**
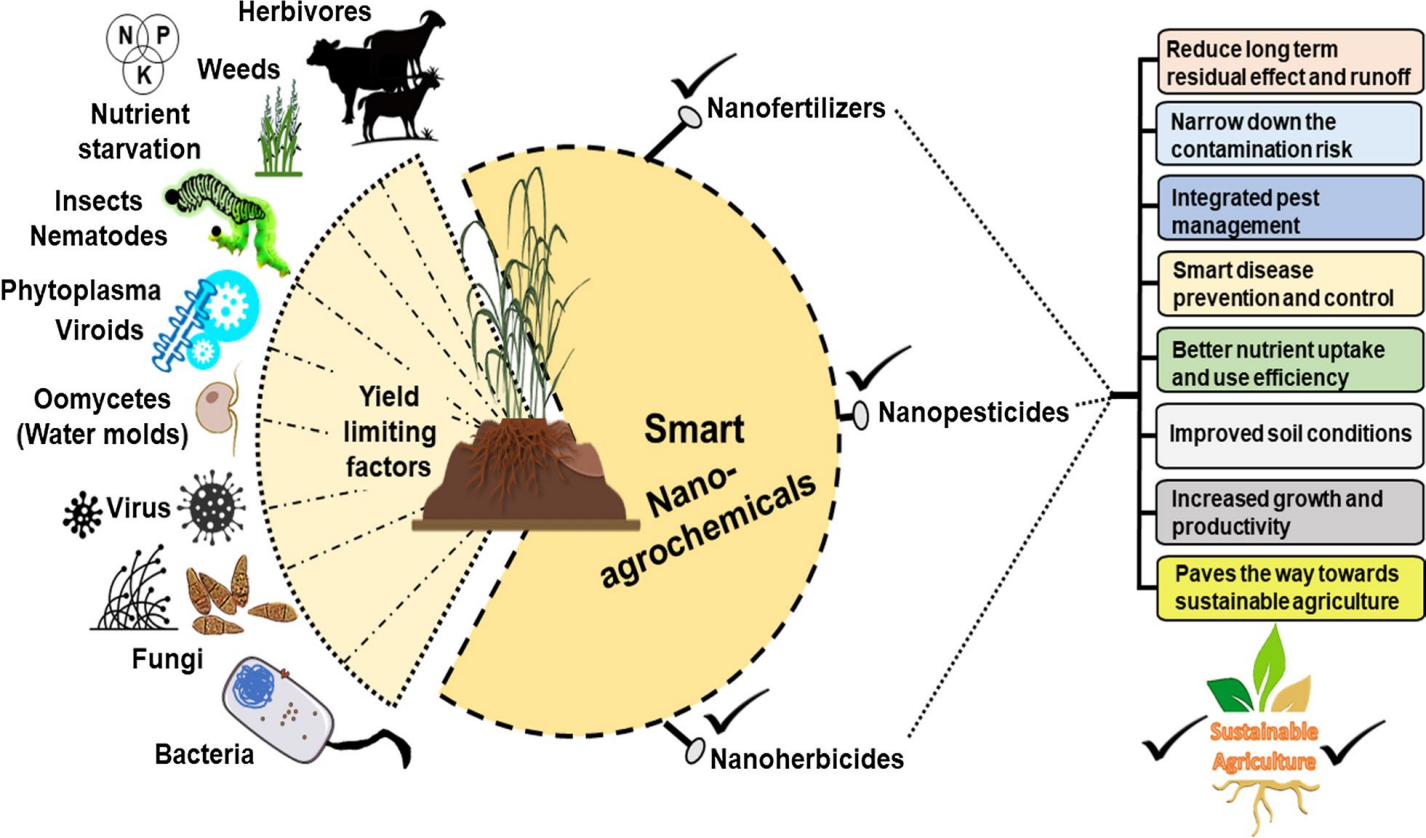
Applications of different nanoparticles for regulation of plant growth, pathogen management, and nutrient uptake in sustainable agriculture

Agrochemicals includes pesticides, herbicides, fungicides, bactericides, nematicides, rodenticides that are used to target pest, weed, pathogenic fungus, bacteria, nematodes and rodents (Fig. 3) [48–50]. Globally, the herbicide market is expanding and is estimated to lies between $27.21 and $39.15 billion at a compound annual growth rate (CAGR) of 6.25% in the expected period 2016–2022. Besides this, the global pesticides market was accounted to reach $70.57 billion by 2021 at a CAGR of 5.15% estimated between 2016 and 2021. Besides this, the global market of encapsulated pesticides grows exponentially at reach benchmark of US $800 million by 2025 expectedly and gains 11.8% CAGR in the tenure of 2019–2025 [18, 19, 48, 49].

The families represented by inorganic chemicals are triazines, phenoxy, and benzoic acid chloroacetanilides representing herbicides, phenylpyrrole, benzimidazoles, dithiocarbamates, and nitriales for fungicide, carbamate, organophosphates, organochlorines relating to insecticide. Smart nanoagrochemicals with nanoformulations must offer a broad variety of benefits including enhanced durability, effectiveness, wettability, good dispersion, less toxicity, good biodegradable ability in soil and environment, and photogenerative nature with the least residues compared to conventional chemicals [51–53]. Over the past, extensive studies were carried out on nanoagrochemicals to access their significant role and contamination range in affecting soil–plant nutrient cycles [19].

### Nanopesticide

The potential utility of nanochemicals in integrated pest management (IPM) depends upon targeted delivery of AIs with increased activity at least drug concentration and proficient monitoring of pesticides interactions with the surroundings. Under harsh conditions, the chemical stability can be achieved by efficient nanocarriers having enhanced dispersal range, wettability, and more protectivity to pesticides without risk of runoff [54–57]. Other noteworthy characteristics of pesticidal nanocompositions can be observed in thermal stability, large surface area, increased target affinity, and biodegradable nature after successful delivery. These delivery systems can be regulated for single goals or multiple combinations viz; spatially target release, time-controlled release, remotely or self-regulated release to overcome the biological barriers in the successful target [21, 58–60]. However, the efficacy of nanoencapsulation or nanocarriers is (1) to prevent pre-degradation of AI in the carrier before their release in the target (2) to improve penetration and ease solubility of AIs within the target site (3) to monitor or regulate the degradation of AIs in the desired site [61, 62].

According to Kremer et al. [63] the adsorptive interaction between pesticides and NPs showing discrete molecular dynamics. Such interactions should have a positive impact on adsorption sites via physiological morphology, binding ability, antioxidant systems, and transportability of pesticides in plants [64]. In *Arabidopsis thaliana*, the antagonistic effect between silver NPs and Diclofop-methyl (post-emergence herbicide) in which herbicides presence decline or affected the Ag^+^ from silver NPs. Moreover, a decrease in pesticide concentration is imperative to avoid their toxicity on non-selected organisms and narrow down contamination risk [65–67]. Several nanocompositions of pesticides have been developed such as nanoemulsions, nanosuspensions, and nanocapsulations. Such nanomaterials are prepared specifically to maintain the regulated release of AIs in several ways including magnetic release, ultrasound release, pH release, heat release, moisture release, DNA-based release, specific release, quick and slow-release [19].

In some cases, nanoparticle delivery in hollow silica NPs are used to prevent avermectin from UV radiation and provide photostability to nanopesticides causes long-term effects on the target organism. Several NPs used various forms of encapsulations including (1) Lipid nanomaterial-based encapsulation. (2) Metal–organic framework-based encapsulation. (3) Polymer-based 6encapsulation. (4) Clay nanomaterial-based encapsulation. (4) Greener encapsulation [9, 42, 43, 45, 47, 68–70].

### Nanofertilizer

Besides plant protection, these smart NPs are extensively used to regulate the physiological process. For example, SiO_2_ NPs (silicon dioxide NPs) elevates seed germination rate in *Lycopersicon esculentum* [71, 72], chitosan-polymethacrylic-NPK increase biomass, nutrient uptake and antioxidant enzymes in *Phaseolus vulgaris* [73, 74], Au-NPs (gold NPs) promotes seed germination, seedling growth, enzymatic activity and nutrient uptake in *Zea mays* [75, 76], SiO_2_-NPs improve uptake of NPK, increase enzymatic activity and seed germination rate in *Hyssopus officinalis* and *Z. mays* [77–79], chitosan-CuNPs (copper NPs) enhance seed germination, activation of α-amylase, protease and activity of various antioxidant enzymes in *Z. mays* [2, 80, 81], chitosan-ZnNPs (zinc NPs) increase accumulation of zinc content and defense enzymes in *Triticum durum* [82, 83], chitosan-γ-polyglutamic acid-gibberellic acid NPs promotes seed germination, root development, leaf area, hormonal efficiency, extracellular enzymes and nutrient efficiency [83, 84], Chitosan-polymethacrylic acid-NPK NPs promotes protein content and nutrient uptake [74, 85], ZnO-NPs (zinc oxide NPs) increase activity of catalase (60.7%), superoxide dismutase (22.8%) and nutrient acquisition [86, 87], CeO_2_-NPs (cerium oxide NPs) enhance seed germination and vigour, enzymatic activity and nutrient uptake in *Spinacia oleracea* and *Z. mays* [88–91], AuNPs increase chlorophyll content and antioxidant enzyme activities in *Brassica juncea* [92] and TiO_2_ NPs (titanium oxide NPs) enhance chlorophyll content, nutrient uptake, activity of Rubisco and antioxidant enzymes in *S. oleracea* and *Cicer arietinum* [89, 93] (Table 1).

**Table 1:** Successful use of nanoformulation used in crop plant as plant growth promoters

Nanoformulation	Mode of applications	Targeted crop	Properties (size/shape/Molecular weight/ pH)	Effect on Plant physiological processes			Key references
				Growth phases	Enzyme activities	Nutrient uptake and release	
SiO_2_ NPs	Seed treatment	*L. esculentum* Mill	12 nm	Enhance seed germination	No visible effect on enzyme activity	–	[71, 72]
Nano-chitosan	Seed treatment	*C. arietinum* L	pH 4.8	Promote total biomass, germination, and vigor index up to (57%)	Increase activities of catalase and superoxide dismutase	–	[83, 170]
Chitosan-polymethacrylic acid-NPK NP	Foliar spray	*P. vulgaris* L	20 nm	Enhance plant growth and total biomass	Enhance the antioxidant enzyme activities	Promote nutrient uptake and accumulation	[73, 74]
Au NPs	Seed imbibition	*Z. mays* L	10–30 nm, spherical	Promote germination and seedling growth	Promote activity of Superoxide dismutase, peroxidase, and catalase	Increased nutrient uptake of maize excluding iron	[75, 76]
SiO_2_NPs	Seed imbibition	*H. officinalis* L	10–20 nm, spherical	Improve plant growth and seed germination	Enhance total soluble proteins	–	[77, 78]
SiO_2_ NPs	Seed Imbibition	*Z. mays* L	10–20 nm, spherical	Promotes seed germination	Enhanced the activities of antioxidant enzymes	Increase uptake of nitrogen, phosphorus, and potassium	[77, 79]
Chitosan-Cu NPs	Seed treatment	*Z. mays* L	Low molecular weight, 80%	Promote seedling growth	Enhance activity of α-amylase and protease	–	[2, 80]
Chitosan-Cu NPs	Foliar spray	*Z. mays* L	50–190 kDa, 80%	Promote seedling growth, overall plant height, and biomass	Enhance activities of defense enzymes	–	[2, 81]
Chitosan-Zn NPs	Foliar spray	*T. durum* L	60 kDa, 85%	Stomatal localization of nanoparticles	Promote defense enzyme activities	Enhance zinc content accumulation by 42%	[82, 83]
Nano-chitosan	Seed treatment	*Z. mays* L	pH 7.0–9.0	Enhance plant height, leaf area, and seed germination	Promote activities of glucose-6-phosphate dehydrogenase, succinate dehydrogenase, and superoxide dismutase	Enhance accumulate of potassium inside the plant	[102, 171]
Chitosan-γ-polyglutamic acid-gibberellic Acid-NPs	Seed treatment	*P. vulgaris* L	290 kDa, 75%–85%, pH 4.5	Promote seed germination, root development, and total leaf area	Enhance the hormonal efficiency, enhance extracellular enzymes, such as cutinase, lipase, and esterase	Increase efficiency of nutrients	[83, 84]
Chitosan-gibberellic acid NPs	Seed treatment	*P. vulgaris* L	27 kDa, 75%–85%, pH 4.5	Promote leaf area, carotenoid and chlorophyll content	Enhance the hormonal efficiency by 90%	Not significant effect on nutrient uptake	[172, 173]
Nano-chitosan	Seed treatment	*P. vulgaris* L	100–399 kDa	Increase seed germination and radical length	Enhance the activity of peroxidase and catalase	Increase Zinc uptake in plant	[174, 175]
Nano-chitosan	Seed treatment	*Capsicum annuum* L	110 kDa, 85–90%, pH 4.0	Enhance the root biomass (77%) and fresh leaf biomass (28%)	Increase the activity of catalase and peroxidase 33% and 23% respectively	Not much significant effect on nutrient uptake	[176, 177]
Chitosan-polymethacrylic acid-NPK NP	Seed treatment	*Pisum sativum* var. *Master B*	20 nm	Enhance mitotic cell division about 1.5 fold	Enhance total soluble proteins like legumin, convicilin and β, vicilin 1, 2 and 3	Enhances the efficiency of plants for the uptake of nutrients	[74, 85]
ZnO NPs	Seed soaked	*Avena sativa* L	20–50 nm, spherical shape	Promote percent germination	–	Modulate uptake of nitrogen and phosphorus in plants	[178, 179]
ZnO NPs	Seed imbibition	*T. aestivum* L	30–40 nm, sphere-crystal	Improve shoot length and total plant biomass	Enhance the activity of superoxide dismutase (22.8%) and catalase (60.7%)	Enhance nutrient acquisition in wheat	[86, 87]
Chitosan-thiamine NP	Seed treatment	*C. arietinum* L	27 kDa, 85%	Promote seed germination and plant growth	Enhance activities of peroxidase, polyphenol oxidase, chitinase, and protease enzyme	Enhance nutrient uptake	[83, 180]
CeO_2_ NPs	50 Mg-Ce per L hydroponic	*Z. mays* L	2–4 nm, crystal	Promote photosynthesis and gas exchange	Accumulation of hydrogen peroxidase enzyme	Increase nutrient uptake of maize	[91, 181]
CeO_2_ NPs	Foliar spray	*S. oleracea* L	4–7 nm	Enhance percent germination (4%) and vigor index	Catalase activity significantly increased	Enhance nutrient uptake	[89, 182]
Nano-chitosan	Foliar spray	*Coffea canephora Piere* var *Robusta*	600 kDa, 85%, pH 6.0	Increase (30–50%) chlorophyll content and photosynthetic rate (30%)	Enhance the enzymatic activities	Enhance nutrient uptake (Nitrogen 10–27%, Phosphorus 17–30% and Potassium 30–45%)	[83]
Chitosan-polymethacrylic acid-NPK NP	Foliar spray	*T. aestivum* L	20 nm	Increase crop yield (50%) and harvest index (24%)	Increase polysaccharides and total saccharides (11%), nitrate reductase enzyme	Accumulation of nitrogen, phosphorus, and potassium in plant	[83]
AuNPs	Spray on leaves	*B. juncea* L. *Czern*	10–20 nm, spherical	Enhance chlorophyll content and plant growth	Enhance antioxidative enzymes, proline and hydrogen peroxide	–	[92]
Carbon (CNTs)	Seeds in culture media	*L. esculentum Mill*	Nanotubes	Enhance seed germination and vigor index	Increase activities of peroxidase, catalase, and superoxide dismutase	Percentage concentration of nutrient elements present in germinated tomato increased	[182]
TiO_2_ NPs	Seeds soaked	*S. oleracea* L	30–60 nm, crystal shape	Enhance seedling growth, biomass, and chlorophyll content	Promote activities of antioxidant enzymes (0.25%)	Enhance nutrient uptake	[88]
TiO_2_ NPs	Spray on leaves	*C.arietinum* L	5–20 nm	Reduce membrane damage during cold stress	Increase activity of Rubisco enzyme	Increase the mineral uptake in plant	[183]

#### Nanoinsecticides

As the trends and demand of encapsulated NPs exponentially increased the regulatory pressure for their management also enhanced simultaneously. Encapsulated insecticides share more than 42% of total pesticide revenue up to 2017 [60, 94, 95]. Recently, in 2019 pesticide manual online classified encapsulated insecticides contain hazardous toxic AIs like pendimethalin, acetochlor, dichlobenil, tefluthrin, etofenprox, chlorpyrifos, carbosulfan, and furathiocarb at the commercial level [19]. The toxicity level of AIs not only depends upon encapsulation material but it helps in adjusting the dynamics of the target species exposure to AIs in vivo conditions [21, 25, 96]. The use of styrene and methylmethacrylate as encapsulation wall material increased the nematicidal activity to suppress the growth of the wheat rust-causing pathogen, *Puccinia reconditea*. Similarly, the effect of urea–formaldehyde and polyuria resin wall on stomatal toxicity, contact toxicity, phoxim loaded microcapsule efficacy, and photolysis properties was reported by Zhang et al. [97]. In another study, improved pest efficiency and poor cytotoxicity of sodium alginate imidachloroprid encapsulation were observed that favored direct application of imidachloroprid [68].

Another study shows a decrease in picloram toxicity to soil microbiota with silica gel encapsulation in comparison to free-form picloform. The silica NPs bioavailability to the non-selected organism can be enhanced by tunning the wall properties of the silica shell [98]. In a study, Jacques et al. [99] reported the atrazine toxicity in encapsulated polymeric and lipid nanocompositions against nematodes*, Caenorhabditis elegans*, but comparably no toxicity was observed in tripolyphosphate/chitosan-based encapsulation that itself can be attributed to low toxicity. Moreover, the oil encapsulated PCL neem-derived nanoencapsulation did not exhibit any adverse effect of stomatal conductance, the photosynthetic ability of maize after exposure up to 300 days. These findings suggest the careful selection of wall material/encapsulation and physicochemical properties of AIs and their composition and application sites [19, 100].

The Si-NPs (silicon NPs) have been efficiently reported to protect infestation from stored beetle *Callosobruchus maculatus* in pulses like *Vigna unguiculata, V. mungo, V. radiate, Macrotyloma uniflorum, C. arietinum,* and *Cajanus cajan* [101]. Despite their excellent performance, nanopesticides show poor commercialization and stability. The pH, temperature, humidity, UV radiation influence AIs availability and influence physiochemical characteristics. Besides these quantity, quality, strict legislation, expensiveness and degradation period of AIs are emerging issues while using nanopesticides [19, 54, 79].

#### Nanofungicides

Beyond the nanocarriers application, nanomaterial as AIs for crop protection is a major aspect of research. The broad spectrum of antifungal properties of nanofungicides can improve their efficiency as a pesticide. For instance, copper, silver, and zinc NPs resolve the disadvantages of chemical AIs for pathogenic resistance with sharp antimicrobial activity and non-toxicity [19]. Moreover, chitosan-based NPs (Ch-NPs) showed effective antifungal activity and restrict growth reported by many research workers in the last decade. For example, Ch-NPs against *Alternaria alternata, Macrophomina phaseolina*, *Rhizoctonia solani* [102]*, Pyricularia grisea, Alternaria solani*, *Fusarium oxysporum* [102, 103]*,* *Pyricularia grisea,* Copper–chitosan NPs against *Fusarium solani* [104], Cu-chitosan NPs- against *R. solani* and *Sclerotium rolfsii* [105], chitosan-saponin NPs [102], oleoyl-chitosan NPs against *Verticillium dahaliae* [106], salicylic acid-loaded chitosan NPs against *Fusarium verticillioides* [107], Ag-chitosan NPs against *R. solani, Aspergillus flavus* and *A. alterneta* [108], silica-chitosan NPs against *Phomopsis asparagi* [109] chitosan-pepper tree (*Schinus molle*) essential oil (CS-EO) NPs against *Aspergillus parasiticus* [110], chitosan boehmite alumina nanocomposites films and thyme oil against *Monilinia laxa* [111] fungicide zineb (Zb) and chitosan-Ag NPs against *Neoscytalidium dimidiatum* [112], chitosan-Thyme-oregano, thyme-tea tree and thyme-peppermint EO mixtures against *Aspergillus niger, A. flavus, A. parasiticus*, and *Penicillium chrysogenum,* [113], chitosan-thymol NPs against *Botrytis cinerea* [39], chitosan-*Cymbopogon martinii* essential oil against *Fusarium graminearum* [114].

In comparison to conventional agrochemicals, the nanoparticle was confirmed to be highly effective in crop protection even at minute concentration viz: 0.43 and 0.75 mg/plate concentration of Ag-doped hollow titanium-oxide (TiO_2_) nanoformulation against Potato pathogens such as *Venturia inaequalis* and *F. solani* [115] (Table 2). Moreover, several successful examples of NPs were studied extensively for abiotic stress tolerance in recent years [116–118]. To cope with drought tolerance, several reports published in past decades on the application of NPs such as TiO_2_ application in *Linum usitatissimum* via elevating pigmentation and reducing the activity of Malondialdehyde (MDA) and Hydrogen peroxide (H_2_O_2_) [119], ZnO promotes effective seed germination in *Glycine max* [120], CuNPs improve pigmentation, biomass and grain yield in *Z. mays* [121]. In case of salinity stress, seed soaking, nutrient solutions, and seed priming methods are used for evaluation in *G. max, S. lycopersicum, and Gossypium hirsutum* respectively [122–124].

**Table 2:** Successful application of nanocomposites for biotic stress tolerance

Pathogen type	Nanoparticles used	Plant disease	Mechanism of action	Key references
*Fungus*				
*Bipolaris sorokiniana*	AgNPs biosynthesized with *Serratia* sp.	Spot blotch pathogen of wheat	Enhance lignification of vascular bundles	[92]
*Gloeophyllum abietinum*	Green-synthesize AgNPs extracted with turnip leaf	Wood-rotting	Inhibit the conidia development	[184]
*Phytophthora capsici*	Ag core-DHPAC shell nanocluster	Blight diseases in Solanaceae	Reduce mycelial growth and sporangial production	[185]
*Escherichia coli, Bacillus* *Subtilis* and *F. oxysporum*	Cu(OH)_2_NPs	Corn leaf blight	Decrease number of conidia	[125]
*F. oxysporum*	Cu_3_(PO_4_)_2_·3H_2_O nanosheets	Root fungal disease in watermelon	Inhibit the fungus growth	[125]
*F. graminearum*	Multiwalled carbon nanotubes, graphene oxide, reduced graphene oxide, and fullerene	Fusarium head blight in wheat	Inhibit spore germination of *Fusarium graminearum*	[96]
*F. oxysporum*	CeO_2_NPs	Panama disease	Enhance antioxidant enzyme activity	[186]
*Aspergillus* spp.	SiNPs	Black mold	Inhibit fungus proliferation	[187]
*R. solani*	Calcium carbonate	Brown rot of stems	Reduce rot growth and recover sucrose level	[188]
*Phytophthora*	Green-synthesize AgNPs extracted with *Artemisia absinthium*	Seed rots	Effect zoospore development	[189]
*Bacteria*				
*X. perforans*	Ag nanoparticles along with graphene oxide	Bacterial spot of tomato	Significantly decrease the activity of *X. perforans*	[190]
*B. sorokiniana*	AgNPs biosynthesized with *Serratia* sp.	Spot blotch pathogen of wheat	Inhibit conidial germination	[191]
*Clavibacter michiganensis*	CuNPs and K_2_SiO_3_NPs	Bacterial ring rot in potato	Decrease bacterial cell viability	[192]
*X. perforans*	Photochemically active TiO_2_NPs	Spot disease in tomato	Due to high photocatalytic activity, reduction in bacterial spot	[193]
*Ralstonia solanacearum*	MgONPs	Vascular wilt disease	Inhibit bacterial activity	[185]
*Xanthomonas campestris* pv. campestris	Silver (Ag) NPs	Bacterial blight	Enhance antioxidant enzyme activity	[194]
*Colletotrichum gloeosporioides*	Chitosan NP	Disease in Chile	Inhibition growth of mycelia	[195]
*A. alternata*	Chitosan NP	Leaf spot	Inhibit spore germination	[83]
*Xanthomonas alfalfae*	Synthesized Mg(OH)_2_NPs	Bacterial leaf spot	Significantly decrease the activity of *X. alfalfae*	[11]

Target species	Nanoparticles used	Mechanism of action	References
*Insects*			
*Aedes aegypti and Anopheles stephensi*	Microbial synthesized Ag, Au, and ZnO-NPs	Epithelial cell, midgut, cortex damage, and thorax shape change	[196]
*Aedes albopictus* and *Culex pipiens pallens*	Ag synthesized using *Cassia fistula* extract	Total protein level, acetylcholinesterase, and α- and decreased activity of ß-carboxylesterase	[197]
*Chironomus riparius*	AgNPs	Modulates GST genes expression, upregulated mRNA expression in delta3, Sigma4 and Epsilon1 GST class	[198]
*C. riparius*	AgNPs	Downregulated activity of ribosomal gene protein, activation of gonadotrophin through upregulation of Balbiani ring protein gene (CrBR2.2) and gonadotrophin-releasing hormone gene (CrGnRH1)	[198]
*C. riparius*	AgNPs	Enhance expression of epsilon-1, sigma-4, and delta-3 and transcript levels of catalase, thioredoxin reductase 1, Mn superoxide dismutase	[198]
*Drosophila melanogaster*	AgNPs	Reduce Cu-dependent enzyme activity, couple with membrane-bound Cu transport protein results in Cu sequestration	[199]
*D. melanogaster*	AgNPs	Cause pigmentation defects and flies locomotive ability	[200]
*D. melanogaster*	AgNPs	DNA-damage, autophagy, ROS-mediated apoptosis	[201]
*D. melanogaster*	Ag and TiO_2_ NPs	Effect developmental processes of flies	[202]
*Sitophilus oryzae*	Nanostructured Al_2_O_3_	Absorbing wax layer that results in insect dehydration	[93]
*Aedes albopictus*	Ag NPs prepared using 3,5-dinitrosalicylic acid and salicylic acid	Total protein, esterase, phosphatase, and acetylcholine esterase enzyme activity decreased	[203]

The application improves stress tolerance by enhancing chlorophyll content, biomass number, soluble sugar content, seed germination [125–127]. According to Shoemaker [128] application of AgNPs (silver NPs) in *Triticum aestivum* increases seedling growth and leaf area whereas foliar application of SeNPs (selenium NPs) improves antioxidant enzyme activity and thylakoid membrane stability in *Sorghum bicolor* under heat stress [129] (Table 3).

**Table 3:** Successful application of nanocomposites for abiotic stress tolerance

Stress type	Nanoparticles	Plant species	Mode of application	Results	Key references
Drought	TiO_2_	*L. usitatissimum* L.	Foliar spray	Enhance chlorophyll and carotenoid content as well as lowers the activity of MDA and H_2_O_2_	[204]
	TiO_2_	*T. aestivum* L. c.v Pishtaz	Foliar spray	Enhance starch content, growth, and yield	[76]
	TiO_2_	*T. aestivum* L.	Amended soil	Increased seedling growth, antioxidant enzymes, total chlorophyll, and carotenoid content	[205]
	ZnO	*G. max* L.	Seed soaking	Enhance germination rate and percent germination	[119]
	Fe_2_O_3_	*Mentha piperita* L.	Hoagland solution	Increase activity of antioxidant enzymes	[206]
	Cu	*Z. mays* L.	Plant priming	Improve plant biomass, chlorophyll, anthocyanins, and grain yield	[207]
	CNTs, graphene	*G. hirsutum* L.	Seed priming	Increase seedling growth and biomass	[123]
	Chitosan NPs	*Hordeum vulgare* L.	Foliar spray	Increase proline content, CAT and SOD	[208]
Salinity	Ag	*Trigonella foenum-graecum*	Seed soaking	Improve percent germination, fresh and dry weight of seedlings	[209]
	ZnO	*Abelmoschus esculentus* L.	Foliar spray	Increase activity of superoxide dismutase, catalase, and photosynthetic pigments	[210]
	ZnO and Si	*Mangifera indica* L.	Foliar spray	Enhance nutrient uptake, carbon assimilation in plants	[211]
	SiO_2_	*Solanum lycopersicum* L.	Seed soaking	Upregulation of stress tolerance genes	[212]
	SiO_2_	*Fragaria ananassa* L.	Soil application	Enhance growth, proline, chlorophyll, epicuticular wax layer and leaf relative water content	[213]
	SiO_2_	*Musa acuminate* L.	Seed priming	Enhance chlorophyll content, shoot growth	[214]
	Ag	*T. aestivum* L.	Seed priming	Enhance total soluble sugars, proline content, and peroxidase activity	[185]
	Fe_2_O_3_	*Helianthus annuus* L.	Foliar spray	Enhance dry weight, leaf area, chlorophyll content	[215]
	Fe_2_O_3_	*Dracocephalum moldavica *L.	Foliar spray	Increase the enzymatic activity of guaiacol peroxidase, catalase, ascorbate peroxidase, and glutathione reductase	[215]
	Mn	*C. annuum* L.	Nanopriming	Improve plant growth	[216]
	CeO	*G. hirsutum* L.	Seed priming	Improve root growth and decrease ROS level	[121]
	CNTs, graphene	*Catharanthus roseus* L.	Murashige and Skoog medium	Enhance the number of leaves and flowers	[124]
	Chitosan-PVA and CuNPs	*S. lycopersicum* L.	Nutrient solution	Enhance chlorophyll, carotenoids, and lycopene content	[122]
Heat	Ag	*T. aestivum* L.	Soil application	Promote the root number, seedling length, and leaf area	[127]
	Se	*S. bicolor* L. *Moench*	Foliar spray	Improve thylakoid membrane stability and activity of antioxidant enzymes	[217]
Heavy metal	Fe	*T. aestivum* L.	Soil application	Increase rate of photosynthesis, chlorophyll content, and plant growth	[87]
UV-B	Si	*T. aestivum* L.	Nutrient solution	Improve antioxidant defense system	[218]
Cold	TiO_2_	*C. arietinum* L.	Amended soil	Decrease MDA levels and electrolyte leakage index	[219]
Flooding	Al_2_O_3_	*G. max* L.	Seed soaking	Increased hypocotyl length, mitochondrial membrane proteins	[220]

#### Nanoherbicide

These NPs inhibit the physiological processes and growth phases in several weed species. For example, Ch-NPs retard germination and growth phases in *Bidens pilosa* [130, 131] NPs atrazine disrupts PSII activity in *Amaranthus viridus* [132], Fe_3_O_4_ NPs (Iron oxide NPs) + purified diatomite + glyphosate decrease pH level in *Cynodon dactylon* [133], Zero valent Fe NPs (Iron NPs) retard germination in *Lolium perenne* [32]. The efficacy of metribuzan, (a commercial herbicide) was enhanced via using NPs to maintain the growth of the weed population including *Melilotus album, T. aestivum, Agrostis stolonifera,* and *Setaria macrocheata* [19]*.*

The atrazine-loaded nanocarriers are used to penetrate the stomatal region, hydathodes and ensure their direct entry into vascular tissues. It ensures the targeting, cellular uptakes, and overcomes intracellular trafficking due to certain properties of NPs: (1) Interaction affinity. (2) Mechanical effect of form and size. (3) catalytic effect. (4) Surface charges/hydrophobicity. Fraceto et al. [19] describing decreased toxicity level of paraquat in non-targeted plants preferring Triphosphate/chitosan nanocarriers application over conventional spray system in *Brassica* sp. Similarly, in *B. pilosa* and *C. dactylon* mortality rate of seedlings was enhanced using encapsulated glyphosate magnetic nanocarriers [19, 131]. The nanoencapsulation uses low doses of herbicide and could effectively reduce the long-term residual effect of herbicides in target species as well as in agricultural land. Conclusively, nanoherbicide can enhance the delivery of AIs in plant tissues and comparatively declined the chance of environmental toxicity [60, 94, 95].

## Impact on plants-soil microbiome

NPs face numerous experience transformation, dissolution aggregation in soil microbiota, adsorption with key regulators that mediate the fate of degradation for organic content, pH, divalent cations, and clay (most important for retention of NPs). According to Asadishad et al. [134], the toxicity of AgNPs depends upon microbial substrate-dependent respiration toward ammonia-oxidizing bacteria decreased with elevation pH content and clay content. Low pH causes the dissolution of AgNPs whereas high soil pH value enhances the negative charge site numbers and leads to increase Ag sorption [19]. In a study, similar results were reported about CuONPs (Copper oxide NPs) on low clay content and organic matter with coarse soil texture. Such acidic soil favors the dissolution of Ag and CuNPs with free ionic liberation, which can elevate the short-duration impact of NPs [9]. Zhai et al. [135] also concluded that nanoformulations of ionic pesticides can show the variable impact, more commonly associated with the fractional ion release. Other authors noted the difference and similarities of ionic and nanoforms of AgNPs with variation in antibacterial activity or the effect on a soil-borne microbial community and their response in in-vitro conditions [19, 136, 137].

In long-term studies, Guilger et al. [66], ensuring routes predictably depend on biogenic NPs, that show the least effect on human cells and denitrification process but are likely to show more impact on plant fungus relationship. At the microscale level, denitrification is a prime microbial activity that gets affected by AgNPs by modulating hydric conditions, pH and creating a devoid zone for fundamental accessories (carbon, nitrate, and oxygen). However, by high soil redox potential value and sandy texture soil favored denitrification, whereas textured clay soils provided offers low redox potential and lies in range for biological transformation [19]. Such impact is correlated by the affinity of AgNPs to denitrification and physicochemical properties ex: surface charge, coating, size, sedimentation rate, dispersibility, and solubility [138]. The biogenic AgNPs are derived from the green process and have no effect on N-cycle reported by Kumar et al. [67]. While the effect of nanocapsules, nanogels, nanometal, and nonmetal particles on soil microbiota as non-selected microbes has been documented. Li et al. [139] evidenced the negative impact of nanopesticide CM-β-CD-MNPs-Diuron complex (carboxymethyl-hdroxypropyl-β-cyclodextrin magnetic NPs) on the activity of the urease enzyme.

The Diuron NPs complex causes declined in the population status of soil bacteria except for actinobacteria with an increase in reactive oxygen species. All these indicate toxicity of CM-β-CD-MNPs-Diuron exert stress on soil microbes and did not reduce even by using Diuron nanoencapsulation [12, 19]. The bionanopesticides treatment was confirmed to improve soil microbiome including weight gain and survival percentages in beneficial earthworm *Eudrilus eugeniae*. It also shows excellent larvicidal, antifeedant, and pupicidal activities against *Helicoverpa armigera* and *Spodoptera* sp. at 100 ppm nanoformulation dose [19, 50, 55].

## Drawbacks using nanoagrochemicals on plants

The nanopesticides are also showing some adverse effects on crop plants directly or indirectly. The most favorable and used AgNPs and their complex nanoparticle have been attributed to their diverse range in each class of pesticides due to low toxicity but still many reported published that explained the drawback of these smart nanoagrochemicals [61, 140, 141] (Table 4). For example, In *Vicia faba*, the AgNPs internalization in leaves can abrupt the stomatal conductance CO_2_ assimilation rate and photosystem II [142]. Furthermore, the binding of AgNPs attaches with Chlorophyll forming a hybrid, that excites electrons 10 times due to fast electron–hole separation and plasmon resonance effect. In another study, AgNPs and AgNPs-graphene oxide GO (Ag@dsDNA GO) effect also observed in *L. esculentum* exhibit antibacterial activity toward *Xanthomonas perforans* [143]. Various reports were submitted in recent years such as ZnO NPs reduced root growth in *Allium cepa* [89], Ch-NPs + paraquat biomass reduction, lipid peroxidation, genotoxicity and leaf necrosis in *Brassica sp.* [144], SiO2NPs affect biomass, germination, protein content, photosynthetic pigment in *Taraxacum officinale* and *Amaranthus retroflexus* [76], AgNPs cause lipid peroxidation, leaf damages and alters catalase activity in *G. max* [145], NPP ATZ + AMZ *Raphanus raphanistrum* suppresses plant growth [146].

**Table 4:** Adverse effect of nanoparticles on targeted crop and soil health

NPs	Size (nm)	Targeted crop	Adverse effect on plant	Degradation time in soil (days)	Effect on soil	Key references
Al_2_O_3_	50	*Nicotiana tabacum* L.	Reduce the germination percentage, biomass per seedling, and average root length	3	Reduce the activity of bacteria *Bacillus cereus* and *Pseudomonas stutzeri*	[147, 148]
C_60-_fullerence	50	*G. max* (L.) Merr	Reduced biomass	60	Reduction of 20–30% in fast-growing protozoa and bacteria	[58, 149]
CuO, Ni, ZnO and Cr_2_O_3_	100	*Oryza sativa* L.	Effect the activities of antioxidant enzymes in plant	24	Activity of enzyme dehydrogenase and urease reduced to 75% and 44% respectively	[221, 222]
ZnO and TiO_2_	10- 20	*T. aestivum* L.	Reduced the root growth by 75%	60	Adversely affect the growth of earthworms, traces of ZnO and TiO_2_ were found inside the body	[61, 223]
Zn^2+^, Zn, and ZnO	50	*Z. mays* L.	50% reduction in photosynthesis, leaf stomatal conductance, transpiration rate, and intercellular CO_2_ concentration	56	Reduce enzymes like β-glucosidase, phosphatase, and dehydrogenase present in the soil	[51, 150]
nZVI (zero valent iron)	20–100	*Salix alba* L.	Effect seedling growth	7	At 750 mg/kg, mortality rate of *Lumbricus rubellus* and *Eisenia fetida* was 100%	[53, 224]
Au	25	*O. sativa* L.	Damage to the root cell wall due to accumulation of Au across xylem	30	Effect the soil microbes and edaphic factors of soil	[52, 225]
TiO_2_, Ag, and CeO_2_	7–45	*A. cepa* L.	Increase in DNA damage as well as lipid peroxidation in roots	14	Reduced the survival, growth and fertility of nematodes	[226]
SnO_2_, CeO_2_ and Fe_3_O_4_	61 (SnO_2_), 50–100 (CeO_2_), 20–30 (Fe_3_O_4_)	*Z. mays* L.	Fe_3_O_4_ results in accumulation of Al in plant roots and negatively affects plant growth	63	Inhibits microbial growth	[141]
Ag	10–20	*P. vulgaris* L.	Disrupt chlorophyll synthesis, nutrient uptake, and hormone regulation	30	50% reduction in the activity of nitrifying bacteria	[158]

Besides these, NPs show an adverse impact on plant physiology, soil microbiota, and declined enzymatic population. For instance; Al_2_O_3_ (Aluminium oxide) reduces bacterial growth and reduces seedling growth [147, 148], C60 fullerene restricts bacterial growth up to 20–30% [149], ZnNPs decrease enzymatic activities in soil and reduces transpiration rate and photosynthetic rate in *Z. mays* [150]. Conclusively, NPs are very reactive and variable in nature, so always a concerning risk for workers who may come across during their application.

## Limitation and challenges at commercial scale implementation

As with documentation, the lack of finding on behavior and fate in the environment of nanoagrochemicals and their impact on faunal diversity may put challenges on their incorporation in agriculture. Instead of the benefits of using nanoencapsulation systems, their implementation requires caution, since it is mandatory to calculate their behavior in the environment and non-targeted communities to develop safer product development policies [54]. Although, it needs to develop smart nanoagrochemicals that are focused on biological nanoformulation and that offer a simple handling process, low cost, more AIs persistence with a sharp release system, and high degradation rate without leaving any residue [148]. Besides these, poor demonstrations at field conditions, cost-effectiveness, consumer acceptance, and feasibility of technology are major constraints on commercial implementation [152].

The limited management guidelines, inconsistence legislative framework, and regulatory models, and lack of public awareness campaign creates inconsistent marketing of such incipient nanoagricultural products. The national and international arrangement that fits at ground level is the only way that supports Nanotechnological development [49]. However, the community seeking approval for nanoagrochemicals must demonstrate the precautionary uses of these new products by proposing unjustifiable safety risks to the user and environment. Thus regulatory guidelines and frameworks are becoming primarily important to resolve the emerging issues of nanoagrochemicals [153]. Moreover, the need for collaboration, discussion, and information exchange forums among countries to ensure threat mitigating strategies should be considered as a milestone in nanoagrochemicals. So consolidates efforts of governmental organizations, scientists, and social communities are needed to preventing the adverse effect of nanoagrochemicals on humans and the environment [59].

In this scenario, the toxicity measuring instrumental setup is used in the characterization of toxicity type and their level to access the potential intrinsic hazards [59]. Currently, the main focus of experimental investigation on nanomaterial translocation in biotic/abiotic systems, monitoring and revealing interaction Among nanotoxicity and nanomaterial in the physical and chemical environment [48, 54, 151–153].

### Transformation

Due to high reactivity, the interaction of nanocomponents with organic and inorganic components in the soil as well as for plants is undetermined and unregulated. The changing in physiochemical properties and transformation behavior after implementation creates chances of heavy metal toxicity. Biotransformation was demonstrated in *Cucumis sativa,* using CeO_2_ bioavailability cause 20% to Ce(III) in the shoots and 15% of Ce(IV) being reduced to Ce(III) in the roots [154]. In another study, AgNPs were oxidized and forming the Ag-glutathione complex in the lettuce plant [154].

### Accumulation of NPs

Because of variability in binding, the accumulation of NPs causes toxicity in plants, humans, and animals. In soybean, CeO_2_ application shut down the Nitrogen fixation cycles and causes toxicity. However, ROS production, growth inhibition, cellular toxicity, and other phytotoxic effect were reported in *Amaranthus tricolor*. The application of C60 fullerene enhanced DDT accumulation in soybean, tomato, and zucchini plants [155].

## Time to switch toward more sustainability

Most agrochemicals are not fully utilized by plants or seep off into the soil, air and water unintendedly causes toxic ill effects and accumulated through biomagnification. Moreover, global pesticide rise threatened biodiversity and led to the adverse effect on human intelligence quotient and fecundity in recent years. Still, it’s also enhancement the resistance in weeds and plant pathogen against agrochemical turn them to super pathogen/weed. New doses after the changing in strategies of pathogens or new strain resurgence enhance cost-effectiveness and put the question on existing regulatory recommendations. [14, 106, 156–158].

The chemicals persist in soil particles, agricultural residues, irrigation water and migrates into the different layers of soils turns into a serious threat to the ecosystem. Leaching of synthetic pesticides, abrupting soil-pest, soil-microbe activities, algal blooms formation, eutrophication, altering soil physiochemical properties [159], and salt toxicity via creating salt buildup in soil [160].

Low-cost oxides of Mg, Al, Fe, Ti, Ce, and Zn (Magnesium, Aluminium, Iron, Titanium, Cerium, Zinc) are ideal candidates and provides greater affinity, a large number of active sites, minimum intraparticle diffusion distance, and maximum specific surface area [160]. NP implementation help to successfully chase down the inorganic residues of various chemicals such as permethrin, 2–4 Dichlorophenoxy acetic acid (2–4-D), Dichlorodiphenyltrichloroethane (DCPT), Diuron (Adsorption), Chlorpyrifos, Chloridazon, Methomyl (Photocatalysis) from the soil. Some nanocomposites are used for complete degradation of lethal agrochemicals for example silver- doped TiO_2_ and gold doped TiO_2_, Zerovalent Fe (nZVI), endosulfan, TiO_2_, nZVI for atrazine, Ag for chlorpyrifos, Pd–Mg, Ni–Fe bimetallic system, nZVI for DDT, nZVI, nitrogen-doped TiO_2_, Fe–Pd (iron–palladium), Fe–S (Iron-sulfur) for Lindane [161] (Table 5).

**Table 5:** Agrochemicals (insecticides, herbicides, and other fungicides) used to regulate the activity of crop pests under a sustainable agriculture approach

Chemical	Trade manufacture company	Nanocomposites used	Crop	Target	Key references
*Insecticides*					
*Inorganic*					
Chlorpyrifos	Dow Chemical Company	PVC	*G. hirustum* L.	*Aphis gossypii*, *Spodoptera frugiperda,* and *Lygus lineolaris*	[227]
	Dow AgroSciences	Chitosan/PLA	*Solanum melongena* L.	*Pseudococcidae*	[228]
Chlorfenapyr	Super Bio Tech Marketing Company	Silica	*Brassica rapa*	*Helicoverpa armigera*	[94]
Avermectin	Super Bio Tech Marketing Company	Polystyrene nanoparticles (PHSN)	*G. hirustum* L.	*Tetranychus urticae*	[229]
	Super Bio Tech Marketing Company	Polydopamine	*Brassica oleraceae*	*Thysanoptera*	[230]
Azadirachtin	Ecobiocides & Botanicals Pvt Ltd	Chitosan	*Ricinus communis* L.	Spodoptera litura	[231]
Deltamethrin	Crop Chemicals India Limited	Chitosan-coated beeswax SLN (Solid–liquid nanoparticles)	*G. hirustum* L., *S. lycopersicum* L.	*Helicoverpa zea, Leucinodes orbonalis*	[185]
Imidacloprid	Chemet Wets & Flows Pvt. Ltd	Sodium alginate	*Nicotiana tobacum* L.	*Cicadellidae*	[232]
Geraniol	Otto Chemie Pvt Ltd	Chitosan/Gum Arabic	*G. hirustum* L.	*Bemisia tabaci*	[233]
Nicotine	Alchem International Pvt. Ltd	Chitosan/TPP	*S. lycopersicum* L.	*Musca domestica*	[234]
*Organic*					
Garlic essential oil	Arishtha Organics Pvt	PEG	*O. sativa* L.	*Tribolium castaneum*	[235]
*A. arborescens* L. essential oil	Priority Biocidal, LLC	SLN	*S. lycopersicum* L.	*Sitophilus zeamais*	[236]
Nanopermethrin	Jerobin J (Hamad medical corporation)	PEG	*–*	*Culex quinquefasciatus*	[237]
Geranium essential oils	India aroma oils and company	PEG	*T. aestivum* L.	*Rhyzopertha dominica*	[238]
Citrus peel essential oil	India aroma oils and company	PEG	*S. lycopersicum* L.	*Tuta absoluta*	[239]
*Rosmarinus officinalis* essential oil	Rosemary essential oil manufacturers & oem manufacturers India	PEG	*O. sativa* L.	*Tribolium castaneum*	[240]
*Herbicides*					
Paraquat	Syngenta	Montmorillonite	*G. max* L.	*Plantago lanceolata* L	[241]
	Syngenta	Chitosan/tripolyphosphate	*Brassica rapa* L.	Soil sorption microalga	[242]
Atrazine	Syngenta	Poly(ε-caprolactone)	*S. bicolor* L.	*Stellaria media* L., *Trifolium repens* L., *Lamium amplexicaule* L	[63]
		SLN	*Brassica napus*	*Raphanus raphanistrum* L	
		Poly (lactic-*co*-glycolic acid)	*Solanum tuberosum* L.	*Croton setigerus* L.*, Oxalis corniculata* L	
Imazapic, Imazapyr	Avansagro chemicals shanghai limited	Alginate/chitosanChitosan/tripolyphosphate	*A. cepa* L.	*B. pilosa* L	[243]
Diuron	Adama Agan Ltd	Chitosan	*Z. mays* L.	*Echinochloa crus-galli* L. *Beauv*	[241]
2,4-D	FirmLimited Company	Nanosized rice husk		*T. repens* L., *Stellaria media* L	
*Fungicides*					
Tebuconazole	Super bio tech	PVP and PVP copolymer	*Pinus taeda* L.	*Gloeophyllum trabeum*	[244]
		Bacterial ghosts	*T. aestivum* L., *H. vulgare* L. and *Cucumis sativus* L.	*Erysiphe graminis* and *Sphaerotheca fuliginea*	
	Super bio tech	Polymeric and SLN	*P. vulgaris* L.	*A. niger*	[11]
Chlorothalonil	Rallis india limited	PVP and PVP copolymer	*Pinus taeda* L.	*Gloeophyllum trabeum*	[244]
Kathon 930	Rohm and haas company	PVC	*Betula alleghaniensis* Britt	*Trametes versicolor*	[245]
Validamycin	Indochem agri science private limited	PHSN	*S. tuberosum* L.	*R. solani*	[246]
Pyraclostrobin	Shijiazhuangdailunchemical co. Ltd	Chitosan–PLA graft copolymer	*Abelmoschus esculentus* L.	*Colletotrichum gossypii*	[247]
		Chitosan/MSN	*S. lycopersicum* L.	*Puccinia asparagi*	[248]
Ferbam	Loveland Products Canada Inc	Gold	*Camellia sinensis*	*Pythium aphanidermatum*	[249]
Carbendazim	Chemet Wets & Flows Pvt. Ltd	Chitosan/Pectin	*Z. mays* L.	*F. oxysporum*	[250]
Prochloraz	Lanfeng biochemical co., Ltd	PHSN	*C. sativus* L.	*B. cinerea*	[251]

## Smart agrochemical: a step ahead toward more sustainability

Al-Barly et al. reported the slow release of nanocomposite fertilizers to depend upon phosphate and nitrogen content availability in soil [162]. TiO_2_ NPs derived from *Moringa oleifera* leaf extract are used to control the red palm weevil (*Rhynchophorus ferrugineus)* and exhibits antioxidant and larvicidal activities. In the case of *Zanthoxylum rhoifolium*, nano-encapsulated essential oil was reported to maintain the population of *Bemisia tabaci* [19, 163]. Nanopesticides derived from pyrethrum insecticides cause an impact on the population status of honey bees. Except for these studies, agrochemical degradation can also be accomplished using adsorption, membrane filtration, catalytic degradation, oxidation, and biological treatment. Since, adsorption using smart Nanosorbents also relies on environmental factors including pH, temperature, and competitive adsorbing molecules [19]. At low pH, the protonated charged active site of NPs disturbs the binding ability of positively charge agrochemical whereas, high temperature creates hinders the electrochemical interactions between active sites and agrochemicals due to elevated vibrate energy of active site of adsorbent and kinetic energy of agrochemicals [79]. Moreover, chitosan-coated and cross-linked chitosan-Ag NPs used as composite microbeads that incorporated into reverse osmosis filters help in the effective removal of atrazine content from the water. According to Aseri et al. [164] integration of membrane filters and magnetic NPs-based beads enhances microbial elimination and resonance activation of water, respectively.

Secondly, targeting a not selected species with possible adverse effect is a key issue emerging that put a loophole of criticism for these smart nanoagrochemicals. For example; 1–10 mg L^−1^ of Polyhydroxybutyrate-co-hydroxyvalerate (PBHA) encapsulation for atrazine in lactuca sativa for 24 h reduced genotoxicity in plants [165], PCL atrazine nanocapsules ill effect on *Daphnia similis* and *Pseudokirchneriella subcapitata,* after exposure up to 24 h [166], Solid lipid NPs encapsulating simazine 0.025–0.25 mg mL^−1^ exhibits *Caenorhabditis elegans* Induction of mortality and decrease in the body length after exposure of 48 h [167]. The uncontrolled non-targeted release of AIs in plant cells causes lysosomal damage with increasing pH. After the cellular compartment, nanoagrochemicals may bind or channelization into cell organelles and causes damage to protein, pigments, and DNA [98].

The binding ability of nanocompositions with selected and non-selected binding helps to recognize its distribution, bioavailability, toxicity level, and exclusion from the plant cell. Several proteins acquire a wide range of functional and structural properties including ligand boding, metabolite production, catalysis, cellular and molecular reorganization [19]. The protein- nanopesticide complex can cause minor structural configuration and denaturation of proteins. Similarly, conformational changes and movement of the genomic DNA mediated through NPs also induced cytogenetic abnormalities. These nanopesticide toxicity are solely dependent upon the balance between key factors like biodegradability, concentration, and size of incorporated AIs. In *Prochilodus lineatus* 20 μg L^−1^ concentration using PCL nanocapsules containing atrazine up to 24–48 h declined toxicity, as they did not induce carbonic anhydrase activity, alterations in glycemia and antioxidant response [168], in *Enchytraeus crypticus* causes a decrease in hatching due to the delayed number of adults and juveniles [19, 158, 169].

No doubt, intervention of nanoagrochemicals, resolve many threats mitigation put forward by the implementation of agrochemical but still more validation is required to lowering the agroecological risks. The persistent use of novel monitoring applications always knocks down the door of improvement of sustainable crop production and protection without creating the threats of NPs as a new contaminant.

## Conclusion and future perspectives

During the entire course of million years of evolution, the green plants had evolved without any interference from other eukaryotes. However, for the last fifty years, continuous human activities have introduced many contaminants in the environment that altered the ecological balance and raised the eye-brows of researchers towards combating the new pathovars and pathotypes. These thrusting biological stresses have severely damaged global crop production. Concerning, the environmental penalty of conventional agrochemicals at present, nanoformulations seem to be a potential applicant for plant protection. The use of controlled biodegradable polymers especially polyhydroxyalkanoates shows significant and attractive properties of biocompatibility, biosorption rate, low-cost synthesis, thermoplastic nature, and ease in biodegradation rate that have popular advantages conventional chemical delivery systems. However, sustainable and efficient utilization with promising target delivery and low toxic effects are prerequisites of commercial implementation. Although, the studies on the soil–plant microbiome and nanoscale characterization highlight the impact of chemical agrochemical on the environment.

The use of nanocoated AIs biopesticides is expected to surpass the challenges of chemical residual management gap and premature degradation of AIs. Instead, these, applying new nanocomponents along with existing chemicals should follow regular checks on resistance strategies of targeted organisms, new resistance pathways, and revolutionized pest strains. Although, smart agrochemicals or nanoagrochemicals resolve so many issues and gives an instant solution.

To ensure these, it is essential to develop more international and national risk assessment, management, and mitigating strategies. Beyond these challenges, social acceptance with reduced environmental cost chiefly soil deterioration, microbiome disruption, depleted water resources need keen monitoring. Ecologically, the continuum uses of agrochemical put the question on survival challenges result in more resistance races creating a vicious loop in which pesticides concentration help to revolutionizing the organism more toward superiority.

For this, alternative strategies with strong monitoring are required, together recommendations of IPM practices help to eliminate shortcomings in individual practices. Despite the advancement in studies on nanoformulation and plant response more extensions in genomic, proteomics, physiological, and metabolic studies help to understand the interaction in the mechanism.

## Data Availability

Not applicable.

## Abbreviations

NPs: Nanoparticles
NMs: Nanomaterils-based products
AIs: Active ingreadents
CRS: Controlled release system
CR: Controlled release
PLA: Poly lactic acid
PLGA: Poly(lactic-co-glycolic acid)
mPEG: Methoxy polyethylene glycol
PCL: Poly(ε-caprolactone
γ-PGA: (Poly (γ-glutamic acid)
γ-GTP: (γ-Glutamyl transpeptidase)
UV: Ultraviolet
PEG: Polyethylene glycol
CAGR: Compound annual growth rate
IPM: Integrated pest management
Ag^+^: Silver
SiO_2_NPs: Silicon dioxide nanoparticles
Ch-polymethacrylic NPK: Chitosan polymethacrylic nitrogen phosphorus potassium
Au-NPs: Gold nanoparticles
ZnO NPs: Zinc oxide nanoparticles
CeO_2_-NPs: Cerium dioxide nanoparticles
TiO_2_NPs: Titanium oxide nanoparticles
*S. oleracea*: *Spinacia oleracea*
Si NPs: Silicon nanoparticles
*V. mungo*: *Vigna mungo*
*V. radiate*: *Vigna radiate*
*C. arietinum*: *Cicer arietinum*
Ch-NPs: Chitosan nanoparticles
CS-EO: Chitosan essential oil
MDA: Malondialdehyde
H_2_O_2_: Hydrogen peroxide
PS II: Photosystem II
Fe_3_O_4_ NPs: Iron oxide nanoparticles
Fe NPs: Iron nanoparticles
*T. aesitivum*: *Triticum aestivum*
*B. pilosa*: *Bidens pilosa*
*C. dactylon*: *Cynodon dactylon*
AgNPs: Silver nanoparticles
CM-β-CD-MNPs-Diuron complex: Carboxymethyl-hdroxypropyl-β-cyclodextrin magnetic nanoparticles diuron complex
Ag@dsDNA GO: Ag@dsDNA-graphene oxide
*L. esculemtum*: *Lycopersicon esculentum*
*Z. mays*: *Zea mays*
CeO_2_: Cerium dioxide
ROS: Reactive oxygen species
Mg: Magnesium
Al: Aluminium
Fe: Iron
Ti: Titanium
Ce: Cerium
Zn: Zinc
2-4-D: 2-4 Dichlorophenoxy acetic acid
DCPT: DDT- Dichlorodiphenyltrichloroethane
nZVI: Zerovalent iron
Fe-Pd: Iron-palladium
Fe-S: Iron-Sulphur
PBHA: Polyhydroxybutyrate-co-hydroxyvalerate
*P. vulgaris*: *Phaseolus vulgaris*
*C. annum*: *Capsicum annum*
*S. oleracea*: *Spinacia oleracea*
*B. juncea*: *Brassica juncea*
CNTs: Carbon nanotubes
Cu_3_(PO_4_)_2_: Copper(II) phosphate
*X. perforans*: *Xanthomonas perforans*
*B. sorokiniana*: *Bipolaris sorokiniana*
*X. alfalfa*: *Xanthomonas alfalfa*
*C. riparius*: *Chironomus riparius*
CrBR2.2: Balbiani ring protein gene
CrGnRH1: Gonadotrophin-releasing hormone gene
*D. melanogaster*: *Drosophila melanogaster*
*L. usitatissimum*: *Linum usitatissimum*
*G. max*: *Glycine max*
SLN: Solid lipid nanoparticles
*G. hirusutum*: *Gossypium hirusutum*
PVA: Poly vinyl alcohol
*S. lycopersicum*: *Solanum lycopersicum*
*S. bicolor*: *Sorghum bicolor*
PVC: Polyvinyl chloride
PHSN: Polystyrene nanoparticles
*O. sativa*: *Oryza sativa*
SnO_2_: Stannic oxide
H. vulgare: Hordeum vulgare
*A. cepa*: *Allium cepa*
*T. repens*: *Trifolium repens*
*H. vulgare*: *Hordeum vulgare*
*S. tuberosum*: *Solanum tuberosum*
MSN: Mesoporous silica nanoparticles
*C. sativus*: *Cucumis sativus*
*B. cinerea*: *Botrytis cinerea*
